# Analysis of bHLH genes from foxtail millet (*Setaria italica*) and their potential relevance to drought stress

**DOI:** 10.1371/journal.pone.0207344

**Published:** 2018-11-09

**Authors:** Pengfei Wang, Haili Wang, Yongmei Wang, Fengshan Ren, Wei Liu

**Affiliations:** 1 Shandong Academy of Grape, Jinan, PR China; 2 Biotechnology Research Center, Shandong Academy of Agricultural Sciences, Jinan, PR China; 3 Shandong Academy of Pesticide Sciences, Jinan, PR China; National Institute of Plant Genome Research, INDIA

## Abstract

Foxtail millet is very a drought-tolerant crop. Basic helix–loop–helix (bHLH) transcription factors are involved in many drought-stress responses, but foxtail millet bHLH genes have been scarcely examined. We identified 149 foxtail millet bHLH genes in a genome-wide analysis and performed Swiss-Prot, GO, and KEGG pathway analyses for these genes. Phylogenetic analyses placed the genes into 25 clades, with some remaining orphans. We identified homologs based on gene trees and Swiss-Prot annotation. We also inferred that some homologs underwent positive selection in foxtail millet ancestors, and selected motifs differed among homologs. Expression of eight foxtail millet bHLH genes varied with drought stress. One of these genes was localized to a QTL that contributes to drought tolerance in foxtail millet. We also perform a *cis*-acting regulatory element analysis on foxtail millet bHLH genes and some drought-induced genes. Foxtail millet bHLH genes were inferred to have a possible key role in drought tolerance. This study clarifies both the function of foxtail millet bHLH genes and drought tolerance in foxtail millet.

## Introduction

The basic helix–loop–helix (bHLH) transcription factor family is a large gene super-family found in plant and animal genomes [1], and its members play very key roles in a wide range of metabolic, physiological, and developmental processes [2–5]. bHLH family members have many different functions [6], and they each contain a core bHLH domain of approximately 60 amino acids, including a basic region (at the N-terminus) and a HLH region [7–8]. bHLH proteins can interact with each other and form homo-dimers or hetero-dimers that are promoted by the bHLH domains [1, 9]. As a core transcription factor domain, the bHLH domain is involved in DNA binding [10], with bHLH domains or bHLH proteins binding to E-box (5′-CANNTG-3′) and G-box (5′-CACGTG-3′) *cis* elements and regulating gene expression [4, 11].

Only a small number of plant bHLH transcription factors have been characterized functionally, far fewer than have been characterized in animals [6]. A previous study showed that bHLH transcription factors can act as transcriptional activators or repressors and are involved in the regulation of fruit dehiscence, anther and epidermal cell development, hormone signalling, and other similar processes in plants [12]. The plant bHLH protein PIF3 is a direct phytochrome reaction partner in the photoreceptor’s signalling network [4] and is involved in controlling the expression of light-regulated genes [13]. Some bHLH transcription factors can interact with MYB transcription factors and WD40 or WDR proteins to form a MYB–bHLH–WD40 (MBW) complex or MYB–bHLH–WDR (MBW) complexes, which can activate anthocyanin biosynthesis genes, resulting in anthocyanin pigment accumulation and fiber development in plants [14–17].

Some functions of unknown bHLH transcription factors as well as some new functions of known bHLH transcription factors have been gradually identified in different plant species. In the medicinal plant *Catharanthus roseus*, the bHLH transcription factor BIS2 is essential for monoterpenoid indole alkaloid production [18]. In *Salvia miltiorrhiza*, bHLH transcription factors are related to tanshinone biosynthesis [19]. *Arabidopsis* bHLH129 appears to regulate root elongation [20], while *Arabidopsis* bHLH109 is associated with somatic embryo induction [21]. Additionally, the *Arabidopsis* bHLH transcription factor PIF4 plays a major role in integrating multiple signals to regulate growth [22]. Research has shown that grasses can use an alternatively wired bHLH transcription factor network to establish stomatal identity [23], further enriching our understanding of plant bHLH transcription factors.

Some plant bHLH transcription factors have also been recently reported to be related to responses to abiotic stresses such as drought and cold. For example, Feng et al. recently found that a novel tomato bHLH transcription factor, SlICE1a, could confer cold, osmotic-stress, and salt tolerance to plants [24]. Similarly, *Eleusine coracana* bHLH57 transcription factors are related to tolerance of drought, salt, and oxidative stresses [25]. bHLH122 plays an important role in drought and osmotic-stress resistance in *Arabidopsi*s [26], where it regulates the expression of genes involved in abiotic stress tolerance [27]. In sheep grass (*Leymus chinensis*), many bHLH transcription factor family members were identified via RNA-seq to be responsive to drought stress [28]. Drought stress could affect plant growth, agricultural yields, and survival. Plants have evolved highly complex reactions to drought stress, and many genes are involved in drought stress [29, 30]. Plant bHLH genes are likely very important in responses to drought stress [29, 30]. Foxtail millet has been proposed as a new model organism for functional genomics studies of the Panicoideae and has the potential to become a new model organism for the study of drought stress responses because of its outstanding tolerance to drought stress [29–31].

We identified the foxtail millet bHLH transcription factors in a genome-wide survey and studied the expression of bHLH genes in foxtail millet in various tissues under drought stress conditions. Our purpose was to identify foxtail millet bHLH transcription factor family members, find candidates that may be relevant to drought stress, and improve the current understanding of drought tolerance mechanisms in foxtail millet.

## Material and methods

### Data collection and identification of bHLH genes

Whole genome sequences of foxtail millet (*Setaria italica*) were obtained from the 2012 Foxtail Millet Database (http://foxtailmillet.genomics.org.cn/page/species/index.jsp) [32]. The bHLH domain is conserved within bHLH proteins, and the HMM ID of the bHLH domain is (PF00010) in the pfam database (http://pfam.xfam.org/). The amino acid sequences of HMMs were used as queries to identify all possible candidate bHLH protein sequences in the foxtail millet genome database using BLASTP (*E* < 0.001). SMART online software (http://smart.embl-heidelberg.de/) was used to identify integrated bHLH domains in putative foxtail millet bHLH proteins. Candidate proteins without integrated bHLH domains were discarded.

### Swiss-Prot, GO and KEGG pathway annotation

We performed Swiss-Prot function annotation analysis based on the UniProtKB/Swiss-Prot database (http://www.uniprot.org/), GO function annotation analysis based on the GO database (http://geneontology.org/page/go-database), and KEGG pathway annotation analysis based on the KEGG database (http://www.kegg.jp/kegg/ko.html).

### Phylogenetic analysis

We aligned the foxtail millet bHLH protein sequences using Clustal Omega online software (http://www.ebi.ac.uk/Tools/msa/clustalo) and constructed neighbor-joining (NJ) trees using MEGA 6.0 with the aligned foxtail millet bHLH protein sequences. Support for inferred evolutionary relationships was calculated from 1000 bootstrap samples [33].

### Conserved motif analysis of foxtail millet bHLH protein sequences

We conducted a conserved Motif Analysis of foxtail millet bHLH protein sequences using the Multiple Em for Motif Elicitation (MEME) suite 4.11.1 software (http://meme.nbcr.net/meme/) [34] with the following parameter settings: output motifs, 20; minimum motif width, 6; maximum motif width, 300 [31].

### Selection pressure analysis

The codeml portion of the phylogenetic analysis maximum likelihood (PAML) program (version 4.7 software) [35] was used to infer potential selective pressures. A comparison of site models M0–M3 was used to determine which kinds of selective pressure the genes underwent, and a M7–M8 comparison was used to identify sites shaped by positive selection [33, 36].

### Identification of foxtail millet bHLH genes within drought tolerance QTLs

QTLs for drought tolerance were identified from previous research by Qie et al [37], and the physical locations of foxtail millet bHLH genes were collected from the foxtail millet genome database (http://foxtailmillet.genomics.org.cn/page/species/index.jsp). The bHLH genes that overlapped with QTLs were inferred to be the genes located within each QTL.

### *Cis*-acting regulatory element analysis

Plantcare (http://bioinformatics.psb.ugent.be/webtools/plantcare/html/) was used to analyse the *cis*-acting regulatory elements of bHLH genes [33].

### Plant material, stress treatments and RNA isolation

To induce drought conditions, 14-day-old foxtail millet cv. ‘Yugu1’ shoots were grown under a 20% polyethylene glycol 6000 (PEG 6000) treatment [29] for 0, 0.5, 6, and 12 h; the 0-h treatment was the control (CK) treatment, while the other treatments simulated droughts of various lengths. The 14-day-old foxtail millet shoots were also grown under a 100 mm/L ABA treatment [38] for 0, 0.5, 6, and 12 h. RNA was isolated using the CTAB method, and we performed reverse transcription according to a previously described protocol [33].

### Gene expression analysis

Quantitative RT-PCR (qRT-PCR) analysis was conducted as previously described [31]. Three replicates were carried out in this study and *t*-tests were used to analyze significance. The qRT-PCR primers are provided in S1 Table. A heat map was generated based on RPKM values using Multiexperiment View software. All the PRKM values or RNA-seq data were based on RNA data hosted by the foxtail millet genome database (http://foxtailmillet.genomics.org.cn/page/species/index.jsp) [32]. RPKM values less than 0.3 were considered unexpressed genes in this study [39].

## Results and discussion

### Identification, annotation, and phylogenetic analysis of foxtail millet bHLH genes

The amino acid sequences of bHLHs were extracted from the foxtail millet genome database (http://foxtailmillet.genomics.org.cn/page/species/index.jsp) using BLASTP with amino acid sequences of bHLH domains (Pfam: PF00010) as queries. We identified 149 bHLH family members distributed among all nine chromosomes. We assayed their annotated functions based on the UniProtKB/Swiss-Prot database (http://www.uniprot.org/). All of these bHLHs were annotated based on the best-hit proteins (S2 Table). In a previous study, the function of some bHLHs from *Arabidopsis* had also been reported [12]. Swiss-Prot functional annotation revealed that most homologs of these *Arabidopsis* bHLHs can be found in foxtail millet excluding some members, including NAI1 (ER body formation); RHD6 and RSL1 (root hair formation); LHW (root development); PRE1, PRE2, PRE3, PRE4, and PRE5 (gibberellin signalling transduction); KDR (light signal transduction); and some orphans.

We also found some functional annotations of bHLH genes that were not identified by a previous study of *Arabidopsis* bHLHs [12], including LAX_ORYSJ transcription factor LAX PANICLE, WIT1_ARATH WPP domain-interacting tail-anchored protein 1, AIB_ARATH transcription factor ABA-INDUCIBLE bHLH-TYPE, MGP_ARATH Zinc finger protein MAGPIE, PP425_ARATH Pentatricopeptide repeat-containing protein, BH032_ARATH transcription factor AIG1, and Anthocyanin regulatory Lc protein (S2 Table).

**Fig 1:**
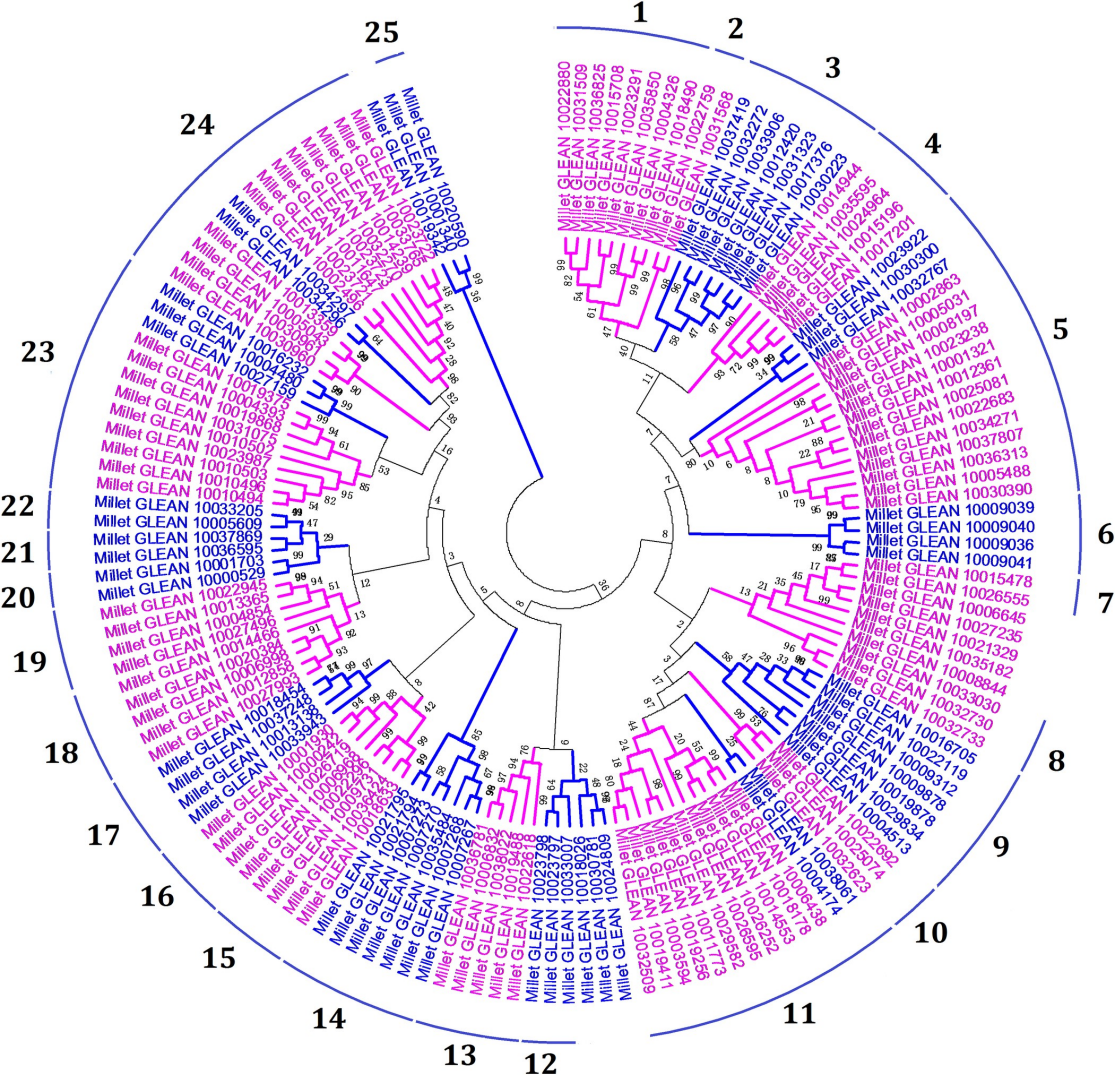
Neighbor-joining phylogenic analysis of bHLHs in foxtail millet. The numbers on the discontinuous cycle represents the names of clades (clades 1–25).

We used the full-length amino acid sequences of the 149 foxtail millet bHLHs for phylogenetic analysis, in which clades with relatively high bootstrap support (≥50) were considered. The phylogenetic tree revealed 25 clades (clades 1–25) in the foxtail millet bHLH family and some orphans (Fig 1). The identified orphan genes were consistent with previous findings by Feller et al., as were the divisions of the clades [12].

The potential function of foxtail millet bHLH genes was studied using gene ontology (GO) annotation analysis, and these bHLH genes were classified into three categories based on GO annotation, cellular component, molecular function, and biological process. Cellular component contains four terms, including nucleus (GO, 0005634), nucleosome (GO, 0000786), intracellular (GO, 0005622), and ribosome (GO, 0005840). Molecular function contained five terms, including amino acid binding (GO, 0016597), DNA binding (GO, 0003677), zinc ion binding (GO, 0008270), structural constituent of ribosome (GO: 000373), and transcription regulator activity (GO, 0030528). Biological process contained three terms, including metabolic process (GO, 0008152), regulation transcription (GO, 0045449), and spermatogenesis (GO 0007283; S3 Table).

Nine foxtail millet homologs of the PIF subfamily members were also found based on Swiss-Prot annotation, and analysis of the KEGG pathway annotation showed that all foxtail millet homologs of PIF subfamily members could be mapped to a KEGG pathway. Millet_GLEAN_10012420 (Swiss-Prot ID, PIF1), Millet_GLEAN_10031323 (PIF5), Millet_GLEAN_10017376 (PIF1), Millet_GLEAN_10030223 (PIF1), and Millet_GLEAN_10009039 (PIF1) were in clade 3 (Fig 1). These genes were mapped to the plant hormone signal transduction pathway (Ko04075) and their KEGG annotation is PIF4 (K16189; S1 Fig). Millet_GLEAN_10031568 (Swiss-Prot ID, PIF3) proteins were in clade 2. These could be mapped onto the circadian rhythm-plant pathway (ko04712), and their KEGG annotation is PIF3 (K12126; S2 Fig). Millet_GLEAN_10009040 (Swiss-Prot ID, PIF1), Millet_GLEAN_10009036 (Swiss-Prot ID, PIF1), and Millet_GLEAN_10009041 (Swiss-Prot ID, PIF1) were in clade 6. These genes were mapped to the plant hormone signal transduction (Ko04075) and circadian rhythm-plant (ko04712) pathways, with corresponding KEGG annotations of PIF4 (K16189) and PIF3 (K12126).

### Selection pressure and motif analysis of foxtail millet bHLH genes

The bHLH genes that were placed into the same clades and had the same annotation categories were considered homologs. We wanted to know if the genes in one homologous group are functionally redundant or functionally divergent, so we performed a selection pressure analysis of some homologous groups. Molecular signatures of selection were categorized as purifying, positive, and neutral. The *d*_n_/*d*_s_ value (ω) can provide a measurement for changes in selective pressures. Values of ω that are equal to, less than, or greater than one indicate neutral evolution, purifying selection, or positive selection on the target genes, respectively [40].

Purifying selection may generate genes with conserved functions or pseudogenization, while neofunctionalization or subfunctionalization is less likely [41]. Signatures of positive selection may indicate adaptive evolution, gene function losses, and pseudogenization [42–44]. Positive selection can also lead to new functions of genes [40, 45–46].

Some homologous groups underwent positive selection, such as the ICE1 group (including Millet_GLEAN_10036424 and Millet_GLEAN_10018633; M0 model, ω (*d*_n_/*d*_s_) = 1.51). The M8vsM7 model showed there were many positive selection sites in the ICE group (BEB analysis, LTR, *P* < 0.1). There was also positive selection in the MYC2 group (including Millet_GLEAN_10010494, Millet_GLEAN_10010503, Millet_GLEAN_10023987, and Millet_GLEAN_10031075; M0 model, ω = 1.85), as shown by the M8 versus M7 comparison (BEB analysis, LTR, *P* < 0.1). As such, we tentatively suggested functional divergence may have occurred in some homologous groups. Moreover, some homologous groups underwent purifying selection, such as the UNE (ω = 0.21), bHLH82 (ω = 0.45) and bHLH35 (ω = 0.26) groups (Table 1).

**Table 1:** Selection pressure of partail homolougus group.

Gene ID	Group ID	ω (dN/dS) of group	Positive site
**Millet_GLEAN_10015708**	**UNE12_ARATH Transcription factor UNE12**	**0.21398**	**18**
**Millet_GLEAN_10023291**			
**Millet_GLEAN_10035850**	**BH082_ARATH Transcription factor bHLH82**	**0.44577**	**9**
**Millet_GLEAN_10004326**			
**Millet_GLEAN_10018490**			
**Millet_GLEAN_10031509**			
**Millet_GLEAN_10010494**	**RAP1_ARATH Transcription factor MYC2**	**1.85238**	**35**
**Millet_GLEAN_10010503**			
**Millet_GLEAN_10023987**			
**Millet_GLEAN_10031075**			
**Millet_GLEAN_10007273**	**BH035_ARATH Transcription factor bHLH35**	**0.25858**	**34**
**Millet_GLEAN_10007270**			
**Millet_GLEAN_10035484**			
**Millet_GLEAN_10007268**			
**Millet_GLEAN_10007267**			
**Millet_GLEAN_10018178**	**BH112_ARATH Transcription factor bHLH112**	**1.50748**	**>100**
**Millet_GLEAN_10026252**			
**Millet_GLEAN_10036424**	**ICE1_ARATH Transcription factor ICE1**	**1.16554**	**>100**
**Millet_GLEAN_10018633**			

**Fig 2:**
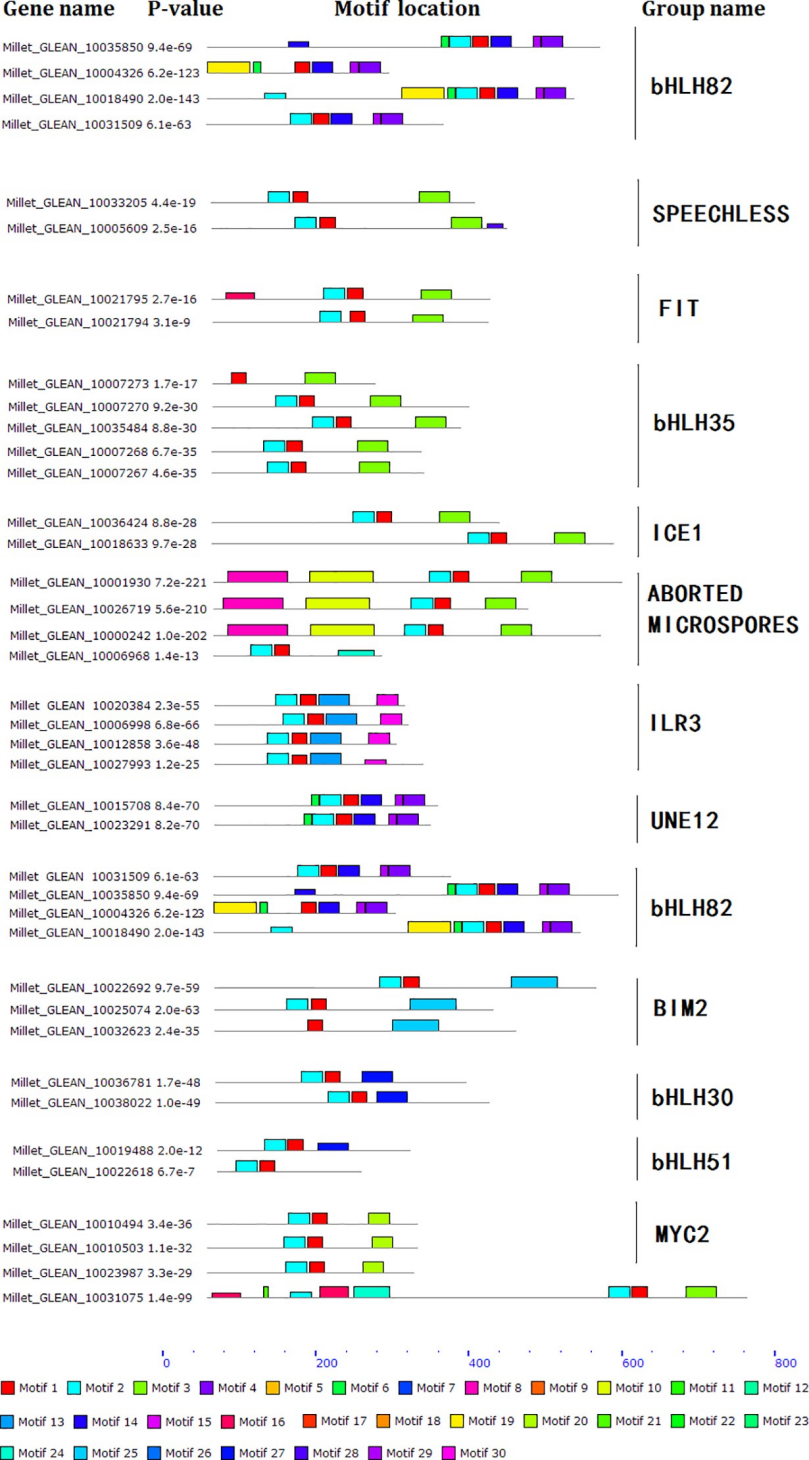
Motifs identified from some bHLH homologous groups in foxtail millet. The names of homologous groups were labeled on right and the name of each gene were labeled on left. Color boxes represent the motifs respectively.

Motif divergence was observed in many homologous groups, such as bHLH82, FIT, bHLH35, BIM2, bHLH51, and myc2 groups. Different motifs may indicate different functions or functional divergence [31]. Motifs of some homologs were in agreement, such as the ILR3, bHLH30, and UNE12 groups (Fig 2). We analyzed the PI, grand average of hydropathicity, instability index, nuclear localization signals, and transmembrane domains of some homologous groups and found functional divergence may also exist in the homologs containing the same motifs. For example, some bHLH30 and ILR3 members contained nuclear localization signals but some did not (S4 Table).

Previous research has indicated that some bHLH genes are duplicated [2, 6]. Duplicated genes are the raw material for the evolution of new biological functions and thus play crucial roles in adaption [47].

### Expression profile of foxtail millet bHLH genes

**Fig 3:**
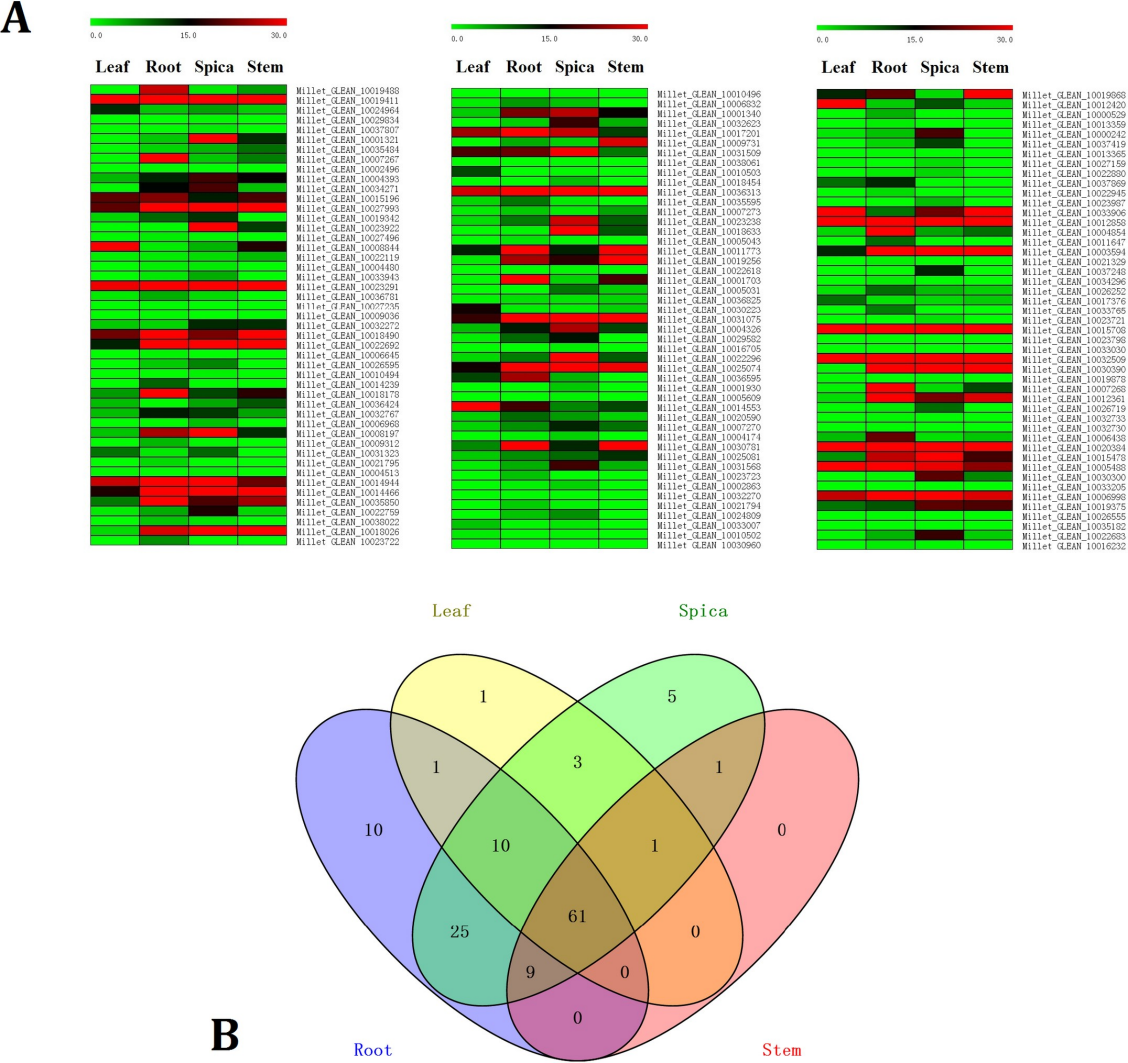
Expression profiles of bHLH genes in different tissues of foxtail millet. A represents the heatmaps of bHLH genes expressions in leaf, root, spica and stem of foxtail millet. B represents The Venn diagram of expressed bHLH genes in four tissues.

The expression profiles of each identified foxtail millet bHLH gene were analysed among several tissues: root, leaf, stem, and spica. The expression levels of foxtail millet bHLH genes in the four tissues based on the previous RNA-seq data (http://foxtailmillet.genomics.org.cn/page/species/index.jsp) and the expression level were captured as RPKM values. Most of these genes were expressed in at least one tissue, and only 20 genes (14.7%) were not expressed in the other three tissues (Fig 3A and S5 Table). According to RPKM values, Millet_GLEAN_10029834, Millet_GLEAN_10037807, Millet_GLEAN_10010494, Millet_GLEAN_10006968, Millet_GLEAN_10018454, Millet_GLEAN_10022618, Millet_GLEAN_10016705, Millet_GLEAN_10001930, Millet_GLEAN_10005609, Millet_GLEAN_10019878, Millet_GLEAN_10023721, Millet_GLEAN_10023987, and Millet_GLEAN_10027159 were only expressed in spica tissue and not expressed in the other three tissues. Millet_GLEAN_10006645, Millet_GLEAN_10014239, Millet_GLEAN_10021795, Millet_GLEAN_10023722, Millet_GLEAN_10023723, Millet_GLEAN_10000529, Millet_GLEAN_10021329, Millet_GLEAN_10033765, and Millet_GLEAN_10034296 were only expressed in root tissue and not expressed in the other three tissues. In contrast, just one gene, Millet_GLEAN_10010503, was only expressed in leaf tissue and not expressed in the other three tissues. No gene was only expressed in stem tissue and not expressed in the other three tissues. The expression of Millet_GLEAN_1002038 (Swiss-Prot ID, ILR3_ARATH transcription factor ILR3) was highest wherever it was expressed in leaf, stem, spica, and root. Its homologs are involved in metal homeostasis, auxin-conjugate metabolism, and salicylic-dependent defence signalling responses in plants [12, 48–49].

In total, 116 foxtail millet bHLH genes were only expressed in root tissue, 77 were only expressed in leaf tissue, 115 were only expressed in spica tissue, and 72 were only expressed in stem tissue. Just 61 genes were expressed in all four tissues (Fig 3B). In contrast, 72 foxtail millet bHLH genes were not expressed in leaf tissue, 33 genes were not expressed in root tissue, 34 genes were not expressed in spica tissue, and 77 were not expressed in stem tissue (Fig 3B and S5 Table). This suggests that foxtail millet bHLH genes are biased towards expression in root and spica tissue.

### Some foxtail millet bHLHs are related to drought stress

Because foxtail millet is remarkably tolerant to drought stress, it has substantial potential to become a new model organism for understanding this trait, which will become even more vital as climate change continues [29]. Previous studies have shown that some plant bHLH genes are involved in tolerance to drought stress [24]. To understand which foxtail millet bHLH members are involved in tolerance to drought stress, candidate genes that are related to drought stress tolerance are first required. In our study, we mainly focused on three kinds of foxtail millet bHLH genes: class A, genes located in QTLs that contribute to drought tolerance; class B, genes whose homologs in other plants were reported to be involved in drought tolerance; and class C, genes that respond to drought stress in foxtail millet.

Six QTLs (LOD > 2.5) for drought tolerance have been identified in foxtail millet, including QGSI_D_7A, QCLD_D_1A, QLRND_D_7A, QCLD_D_1B, QCLR_D_6A, and QSR_D_1A [37]. In this study, we determined that only the QTLs QGSI_D_7A (chr7, 33,221,000–27,196,000), QCLD_D_1A (chr1, 29,834,000–32947000), and QLRND_D_7A (chr7, 30,571,000–21,648,000) contain bHLH genes in foxtail millet. QTL QCLD_D_1A is related to coleoptile length decreases in foxtail millet [37]. This QTL was estimated to contribute 7% of the observed phenotypic variance [37], and it also contained the bHLH genes Millet_GLEAN_10023797 (Swiss-Prot ID, bHLH95), Millet_GLEAN_10023798 (Swiss-Prot ID, bHLH95), and Millet_GLEAN_10035595 (Swiss-Prot ID, bHLH128). QTL QLRND_D_7A was related to a lateral root number decrease of foxtail millet [37]. It was estimated to contribute 10% of the observed phenotypic variance [37], and it contained the bHLH genes Millet_GLEA N _1 00 02496 (Swiss-Prot ID, bHLH25) and Millet_GLEAN_10029582 (Swiss-Prot ID, bHLH113). QTL QGSI_D_7A was related to the germination stress tolerance index [37]. It was estimated to contribute 14% of the phenotypic variance [37], and it contained the bHLH genes Millet_GLEAN_10016232 (Swiss-Prot ID, ARLC_MAIZE Anthocyanin regulatory Lc protein), Millet_GLEAN_10037248 (Swiss-Prot ID, bHLH91), and Millet_GLEAN_10002496 (Swiss-Prot ID, bHLH25, which is also contained by the QTL QLRND_D_7A) (Table 2).

**Table 2:** Foxtail millet bHLH genes in QTL for drought tolerance.

			Percentage of	
QTL name	Chr.	Marker interval	the variance explained	bHLH genes in QTL
**QTL QCLD_D_1A**	**1**	**p88-p16**	**7%**	**Millet_GLEAN_10023797**
				**Millet_GLEAN_10023798**
				**Millet_GLEAN_10035595**
**QTL QLRND_D_7A**	**7**	**si136-si119**	**10%**	**Millet_GLEAN_10002496**
				**Millet_GLEAN_10029582**
**QTL QGSI_D_7A**	**7**	**si256- sims1409**	**14%**	**Millet_GLEAN_10016232**
				**Millet_GLEAN_10037248**
				**Millet_GLEAN_10002496**

Currently, some plant genes, including bHLH57 [25], bHLH 122 [26], bHLH112 [27], ABA-Inducible bHLH or MYC2 [43–44], and ICE [24, 50–51], have been reported to be involved in drought tolerance. In our study, we were unable to identify the homologs of bHLH122 and bHLH57 in foxtail millet, but we did find the homologs of bHLH112, ABA-Inducible bHLH or MYC2, and ICE based on the Swiss-Prot annotation, including Millet_GLEAN_10026252 and Millet_GLEAN_10018178 (Swiss-Prot ID, bHLH112).

By referring to previous RNA-seq data [29], we found eight foxtail millet bHLH genes were involved in the response to drought stress (i.e., the 20% PEG 6000 treatment). Most class A and B genes did not respond to drought conditions, except for Millet_GLEAN_10035595 (Swiss-Prot ID, bHLH128; the function of bHLH 128 is unknown). The other seven genes that did respond were Millet_GLEAN_10023721 (Swiss-Prot ID, bHLH25), Millet_GLEAN_10007270 (Swiss-Prot ID, bHLH35), Millet_GLEAN_10008844 (Swiss-Prot ID, UNE10; UNE10 is involved in the fertilization process), Millet_GLEAN_10005488 (Swiss-Prot ID, factor bHLH49), Millet_GLEAN_10036595 (Swiss-Prot ID, ORG2; ORG2 is involved in Iron homeostasis), Millet_GLEAN_10007267 (Swiss-Prot ID, bHLH35), and Millet_GLEAN_10030390 (Swiss-Prot ID, bHLH49). Excluding Millet_GLEAN_10008844 and Millet_GLEAN_10036595, the function of homologs from other plant species of the other six genes were unknown. Additionally, the identified function of homologs from other plant species, Millet_GLEAN_10008844 and Millet_GLEAN_10036595, are not thought to be involved in tolerance to drought stress, as shown by a previous study [12].

**Fig 4:**
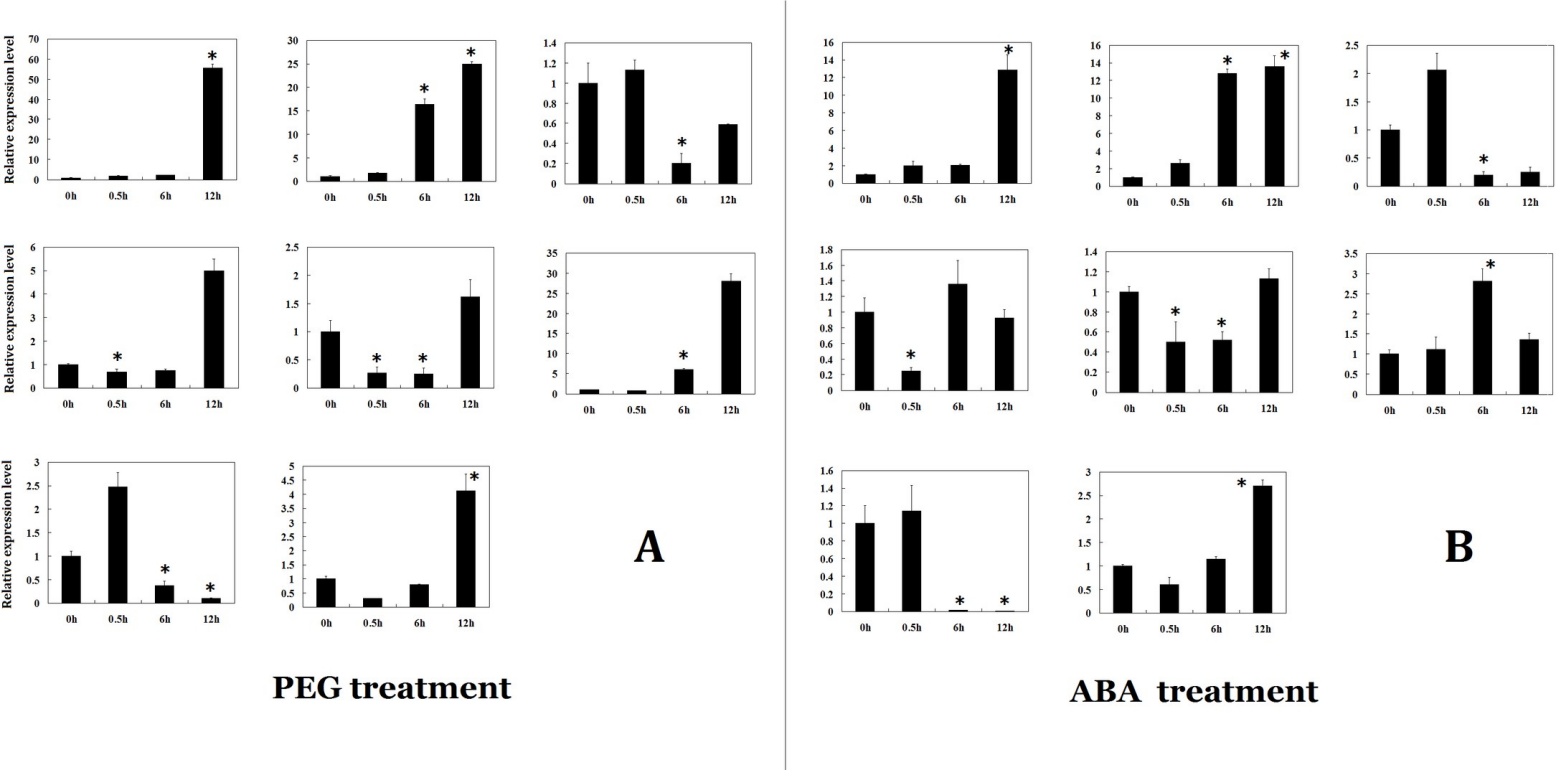
Expression patterns of eight bHLH genes under PEG or ABA treatment. A represents the relative expression levels of eight bHLH genes under PEG treatment. B represents the relative expression levels of bHLH genes under ABA treatment. Y axis represents the relative expressed level. X axis represents the time-points of treatment. “*” represent significantly changed comparing with that of CK (0h) (p<0.05).

We analyzed the expressions of the genes that respond to drought stress using qRT-PCR. The 14-day foxtail millet shoots were subjected to 20% PEG 6000 for 0, 0.5 h, 6 h and 12 h treatment. The expression levels of the eight genes treated for 6 h and 12 h under PEG were significantly changed, and they all showed similar variation trends with that of the Qi’s RNA-seq data excluding Millet_GLEAN_10036595 (Fig 4A) [29].

Many candidate genes among the foxtail millet bHLH members have been linked to drought stress tolerance in other species. Accordingly, bHLH genes are likely to play an important role in drought stress tolerance in fox millet, though the homologs in other species of some candidates were not determined to be involved in tolerance to drought stress by previous studies, such as the homologs of bHLH128, bHLH35, bHLH25, and ORG2. We guessed these candidates (homologs of bHLH128, bHLH35, bHLH25 and ORG2) may have evolved different function from their homologs in foxtail millet or other species, and their functions may have contributed to drought stress tolerance, though perhaps they were not assayed clearly. Our inference is based on the analysis of sequence motifs and a molecular signature of selection. Divergence in motifs and positive selection between homologs or duplication pairs suggested functional divergence may have occurred in these gene trees while genes with de novo functions were created through expansion of the foxtail millet bHLH family.

### The role of bHLH in foxtail millet drought resistance

ABA-dependent and -independent signalling pathways appear to be involved in drought stress tolerance. However, previous studies ignored a direct link between AREB and bHLH (such as ICE and Myc) in tolerance to drought stress tolerance. Rather, bHLH was only considered part of the DREB/CBF pathway, which may be involved in tolerance to drought and cold stress [52]. However, analysis of candidate promoters showed that most contained AREB elements (*cis*-acting elements involved in abscisic acid responsiveness; S6 Table). This research showed these candidates may be regulated by AREB. The 14-day foxtail millet shoots were subjected to exogenous ABA treatments of 0, 0.5, 7, and 12 h. The changes in expression of the eight genes under the ABA treatment were similar to the changes in expression of the eight genes under the PEG treatment (i.e., drought stress) over the same treatment times (Fig 4B). This indicated that these foxtail millet bHLHs genes may be regulated by ABA and that drought may regulate these genes through the ABA-dependent signalling pathway. Analysis of promoters revealed that most promoters of the candidate genes we identified contain MBS elements (MYB binding sites involved in drought inducibility; S6 Table) and some contain G-box element. Accordingly, MYB genes may regulate bHLH genes, and bHLHs may in turn regulate drought responses.

Additionally, we comprehensively identified the promoters of 40 drought-responsive genes in common between foxtail millet and some monocot and dicot species [29, 53–55] including the drought-responsive marker gene COR47 [29], which contains E-box and G-box elements (S7 Table). These genes may accordingly be regulated by bHLH genes. Thus, we propose the following hypothesis. When foxtail millet is under drought stress, some foxtail millet bHLH genes may be regulated by ABA-dependent signalling pathways, including AREB, MYB, or bHLH transcript factors; it is these genes that could affect downstream genes that are directly involved in drought stress responses.

## Supporting information

S1 Fig Roles of bHLHs in foxtail millet hormone signal transduction pathway. Red boxes represents the location of foxtail millet bHLHs.
(TIF)

S2 Fig Roles of bHLHs in foxtail millet circadian rhythm pathway. Red boxes represents the location of foxtail millet bHLHs.
(TIF)

S1 Table qRT-PCR primers for bHLH genes in foxtail millet. (XLS)

S2 Table bHLH genes and function annotation in foxtail millet. (XLS)

S3 Table GO annotation of bHLH genes in foxtail millet. (XLS)

S4 Table Physicochemical properties of some homologous groups. (XLSX)

S5 Table RPKM value of bHLH genes in foxtail millet tissues. (XLS)

S6 Table Cis-acting elements of candidate bHLH promoters in foxtail millet. (XLS)

S7 Table Cis-acting elements of drought-responsive gene promoters in foxtail millet. (XLS)
