# Supplementary figures and images for: Analysis of bHLH genes from foxtail millet (Setaria italica) and their potential relevance to drought stress
Source: PLoS One. 2018 Nov 9;13(11):e0207344. doi: 10.1371/journal.pone.0207344 (PMC6226204; doi:10.1371/journal.pone.0207344)

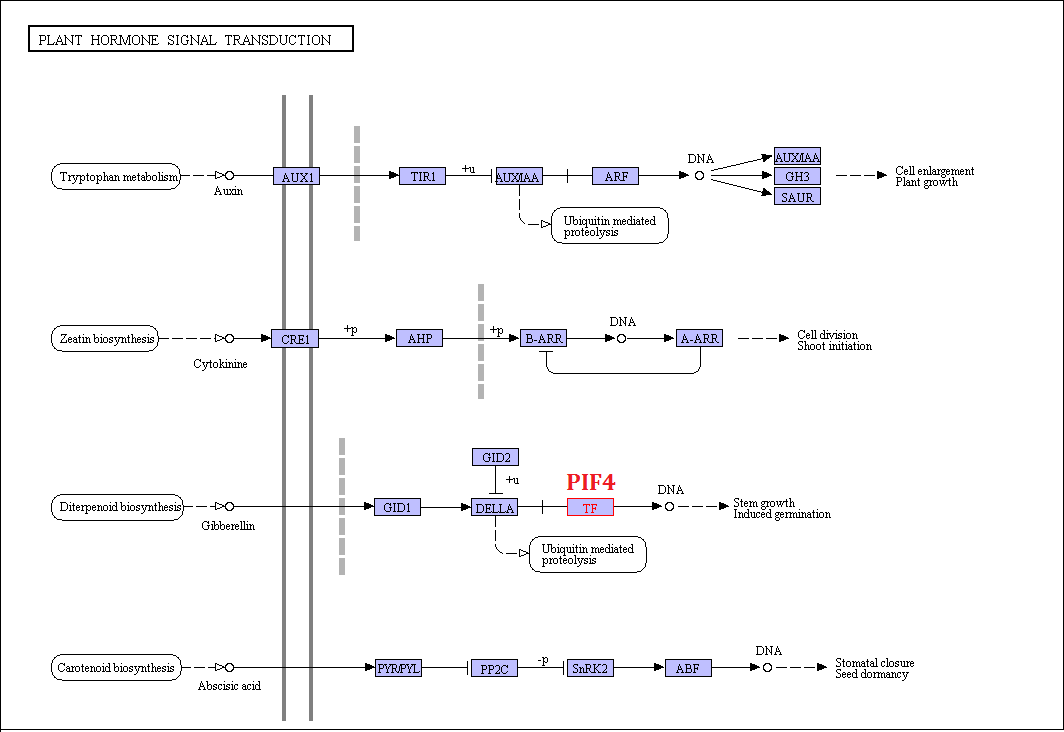

Supplement: S1 Fig — Red boxes represents the location of foxtail millet bHLHs. (TIF) [file pone.0207344.s001.tif]

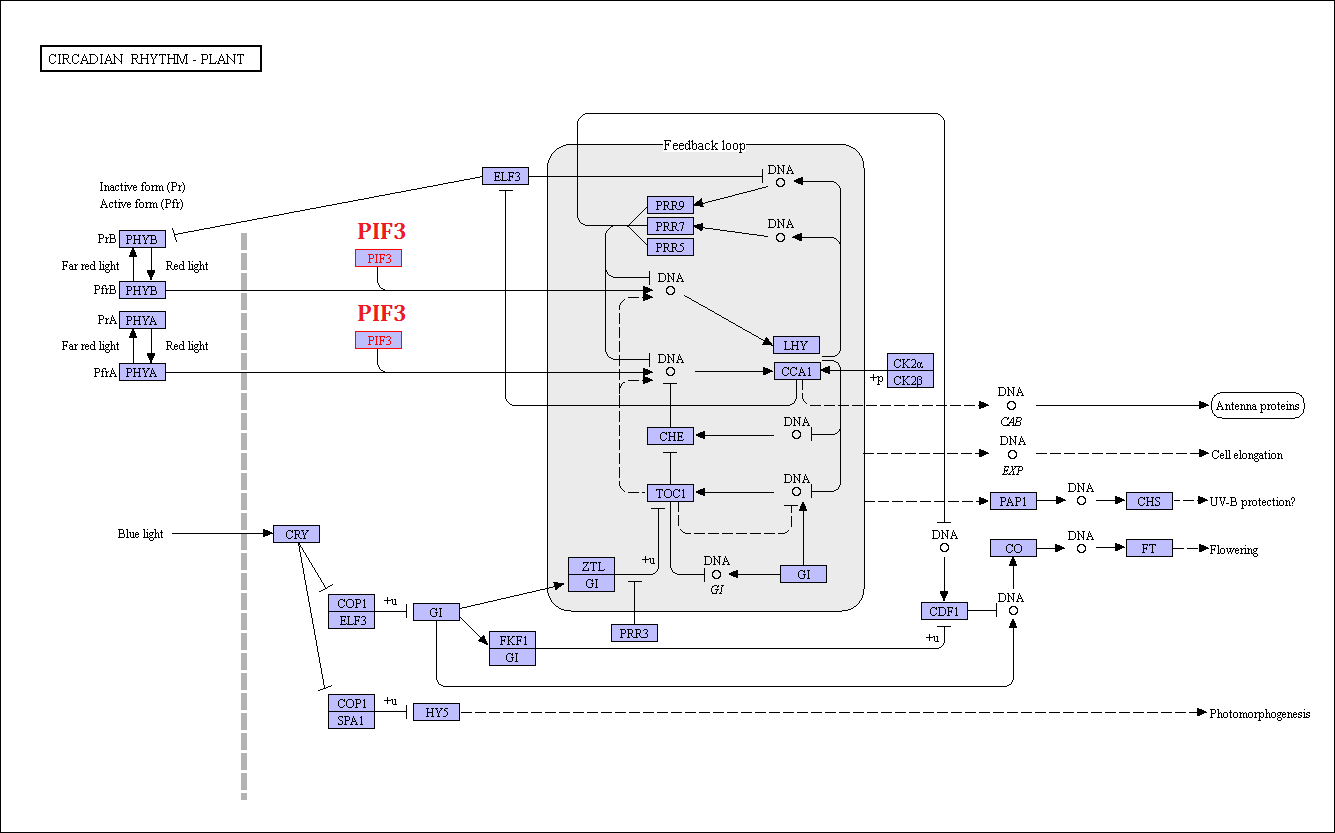

Supplement: S2 Fig — Red boxes represents the location of foxtail millet bHLHs. (TIF) [file pone.0207344.s002.tif]
